# Postoperative Bleeding Definitions as the Foundation of Hemostasis in Pediatric Cardiac Surgery

**DOI:** 10.3390/diagnostics16091375

**Published:** 2026-05-01

**Authors:** Oliver Karam, Christie Atchison, Madhuradhar Chegondi

**Affiliations:** 1 Pediatric Critical Care Medicine, Yale School of Medicine, 333 Cedar St, New Haven, CT 06520, USA; 2 Pediatric Critical Care Medicine, Emory University School of Medicine, Children’s Healthcare of Atlanta, Atlanta, GA 30329, USA; christie.atchison@choa.org; 3 Pediatric Critical Care Medicine, Children’s Hospital of Illinois, University of Illinois College of Medicine, Peoria, IL 61637, USA; chegondi@uic.edu

**Keywords:** bleeding, hemostasis, benchmarking, clinical relevance, electronic health records, decision making, child, infant, neonate

## Abstract

Effective perioperative hemostasis in pediatric cardiac surgery depends not only on accurate diagnostics and targeted transfusion strategies but also on a clear and consistent definition of postoperative bleeding. Despite the clinical importance of bleeding in neonates and children undergoing cardiopulmonary bypass, bleeding remains variably defined across institutions, registries, and clinical trials. This heterogeneity complicates bedside decision-making, limits benchmarking, and weakens the interpretation of interventional studies. In this review, we examine postoperative bleeding definitions as a foundational component of hemostatic management in pediatric cardiac surgery. We summarize commonly used adult bleeding definitions and highlight their variability and limited applicability to neonatal and infant physiology. We review current pediatric approaches, including chest tube output-based thresholds and multidimensional severity scales that incorporate clinical impact and physiologic consequences, while avoiding reliance on transfusion or procedural interventions alone. We discuss the limitations of intervention-driven criteria, the challenges of quantifying blood loss, and the influence of developmental hemostasis and surgical complexity. We also explore structural barriers within electronic medical records that impede standardized data capture and consider harmonization efforts in ECMO populations as a potential model. By outlining the consequences of definitional heterogeneity and proposing principles for standardization, this manuscript aims to support more consistent hemostatic care, meaningful benchmarking, and stronger multicenter research in pediatric cardiac surgery. We recommend that future multistakeholder consensus efforts develop a multidimensional, developmentally calibrated bleeding definition that integrates quantitative blood loss, physiologic impact, and clinical consequences while clearly separating bleeding severity from the interventions used to treat it.

## 1. Introduction

Perioperative hemostasis and transfusion management in pediatric cardiac surgery require precise diagnostics, thoughtful clinical judgment, and coordinated multidisciplinary care. Central to this process is the ability to identify and classify postoperative bleeding in a meaningful and reproducible way. Bleeding remains a frequent and clinically significant complication in neonates and children undergoing cardiopulmonary bypass, influencing transfusion exposure, reintervention, organ dysfunction, and survival.

Despite its importance, postoperative bleeding is defined inconsistently across pediatric centers, registries, and clinical studies. While adult cardiovascular medicine has proposed several structured bleeding definitions, even these definitions vary in their criteria and agreement. In pediatric cardiac surgery, no universally accepted, developmentally calibrated definition exists. Current approaches rely on heterogeneous combinations of chest tube output, transfusion exposure, laboratory changes, and procedural interventions.

This narrative review examines postoperative bleeding definitions as a foundational element of perioperative hemostasis and explores pathways toward greater standardization in pediatric cardiac surgery. Sources were identified through targeted PubMed searches and the reference lists of recent consensus publications on bleeding definitions in pediatric cardiac surgery, pediatric critical care, and mechanical circulatory support, supplemented by the authors’ topic expertise; no formal inclusion criteria or quality assessment were applied. We do not propose a new authoritative definition, as this would properly emerge from a multistakeholder consensus process. Our contribution is instead threefold: to compare existing adult and pediatric bleeding definitions in a unified format, to identify the structural limitations that constrain current definitions (particularly the embedding of clinician-driven responses within the definition itself), and to outline principles that future consensus efforts could build on.

## 2. Why Definitions Matter

Perioperative management of hemostasis and transfusion in pediatric cardiac surgery spans a continuum, from diagnostic assessment of coagulation to targeted transfusion, pharmacologic intervention, and surgical decision-making. This continuum, however, depends on a fundamental and often underappreciated element: a clear definition of postoperative bleeding. Without consensus on what constitutes clinically significant bleeding, even the most advanced diagnostic tools and transfusion algorithms lack a stable foundation.

Postoperative bleeding directly shapes transfusion exposure, need for reintervention, duration of mechanical support, organ dysfunction, and survival in neonates and children undergoing complex cardiac procedures [1]. Yet bleeding remains variably defined across institutions, registries, and clinical trials. Clinicians rely on differing chest tube output thresholds, transfusion volumes, or decisions to return to the operating room to categorize severity [2]. When definitions incorporate the need for transfusion or reoperation, clinician behavior becomes embedded within the outcome, particularly in environments where transfusion triggers and surgical thresholds vary.

In research, heterogeneous bleeding endpoints limit comparability and dilute the interpretability of interventional trials evaluating antifibrinolytics, coagulation factors, or viscoelastic-guided transfusion strategies. These challenges are magnified in pediatrics, where small circulating volumes, developmental hemostasis, cardiopulmonary bypass exposure, and lesion complexity create profound physiologic variability. A unified, developmentally appropriate definition of postoperative bleeding is therefore foundational to strengthening perioperative hemostatic care and advancing the science of pediatric cardiac surgery.

## 3. Adult Bleeding Definitions

In contrast to pediatrics, adult cardiovascular medicine has benefited from several consensus bleeding definitions that have strengthened comparability across trials and informed clinical practice (Table 1). Definitions such as those from the International Society on Thrombosis and Haemostasis (ISTH), the World Health Organization (WHO) bleeding scale, and the Bleeding Academic Research Consortium (BARC) introduced structured, graded definitions based on clinical impact, laboratory changes, transfusion requirements, and need for procedural intervention. These definitions improved endpoint consistency in trials evaluating antithrombotic therapy, cardiac surgery outcomes, and percutaneous coronary interventions.

The ISTH major bleeding definition for patients on anticoagulation includes bleeding that is: (1) fatal, (2) occurs in a critical site (intracranial, intraspinal, intraocular, pericardial, retroperitoneal, intramuscular with compartment syndrome), (3) causes a hemoglobin decrease ≥2 g/dL, or (4) requires transfusion of ≥2 units of blood [3].

In adult cardiac surgery, bleeding severity is typically characterized using structured combinations of quantitative blood loss, transfusion exposure, and need for surgical reintervention. The BARC Type 4 definition was specifically developed to standardize bleeding outcomes following coronary artery bypass grafting [4]. It classifies bleeding as major if it includes perioperative intracranial hemorrhage within 48 h, reoperation after sternal closure for the purpose of controlling bleeding, transfusion of five or more units of whole blood or packed red blood cells within 48 h, or chest tube drainage of at least 2 L within 24 h. These criteria illustrate an approach in adults of anchoring bleeding severity to both measured blood loss and clinically consequential interventions.

The TIMI (Thrombolysis in Myocardial Infarction) bleeding definition is a laboratory-based system that relies primarily on hemoglobin/hematocrit changes [5]. TIMI Major Bleeding is defined as (1) intracranial hemorrhage, or (2) hemoglobin decrease ≥5 g/dL (or hematocrit decrease ≥15%), with or without an identified bleeding site.

The ISTH and BARC definitions show substantial agreement (Cohen’s κ = 0.69) in identifying major bleeding cases. Meanwhile, TIMI definitions show poor agreement with both ISTH and BARC major bleeding definitions (Cohen’s κ = 0.24 and 0.22, respectively) [11].

## 4. Current Pediatric Bleeding Definitions

Although adult definitions provide a useful conceptual starting point, their direct application to pediatric cardiac surgery is problematic. Adult criteria frequently rely on absolute transfusion volumes or fixed laboratory thresholds that do not scale appropriately to neonatal or infant physiology. A transfusion volume considered “major” in adults may represent a small fraction of circulating blood volume in that population, yet the same absolute threshold would be catastrophic in a neonate. Small circulating volumes, developmental differences in coagulation, and the near-universal exposure to cardiopulmonary bypass in congenital heart surgery create hemostatic dynamics that differ fundamentally from those in adult practice. In addition, transfusion triggers and thresholds for reoperation vary substantially across pediatric centers, further complicating the use of intervention-based definitions.

Despite these challenges, pediatric cardiac surgery lacks a universally adopted bleeding definition. Most studies rely on center-specific chest tube output thresholds, commonly expressed in mL/kg/hour, yet the selected cutoffs and time intervals vary widely [2]. Others define bleeding based on transfusion volume within a defined postoperative window or the need for return to the operating room for bleeding control. For example, the validated pediatric-specific definition developed by Bercovitz et al. defines excessive bleeding meeting any one of the following criteria: (1) chest tube output ≥7 mL/kg/h for ≥2 consecutive hours in the first 12 postoperative hours, (2) chest tube output ≥84 mL/kg total for the first 24 postoperative hours, or (3) surgical re-exploration for bleeding or cardiac tamponade physiology in the first 24 postoperative hours [6].

The Bleeding Assessment Scale in Critically Ill Children (BASIC) definition is worth mentioning, although it is not specific to cardiac surgery. It incorporates multiple domains of bleeding severity, including clinical impact and physiologic consequences, and avoids relying solely on transfusion volume or procedural intervention as defining criteria [7]. By emphasizing observable bleeding characteristics and patient-centered effects rather than clinician-driven responses, BASIC offers a behavior-independent definition.

Current pediatric bleeding definitions, therefore, remain fragmented (Table 1) and inconsistently applied across centers. The absence of a standardized, developmentally calibrated definition continues to limit benchmarking, multicenter collaboration, and the design of rigorous interventional trials in pediatric cardiac surgery.

## 5. Unique Pediatric Physiologic and Surgical Factors

Any effort to define postoperative bleeding in pediatric cardiac surgery must account for the unique physiologic and surgical context of this population. Neonates and infants have markedly smaller circulating blood volumes [12,13], such that even modest absolute blood loss may represent a substantial percentage of total volume. A drainage rate that appears numerically small may therefore have profound hemodynamic and metabolic consequences.

Developmental hemostasis further distinguishes pediatric patients from adults. Age-dependent differences in coagulation factor levels, platelet function, fibrinolytic activity, and endogenous anticoagulant pathways create a baseline hemostatic profile that evolves over the first months and years of life [14].

Cardiopulmonary bypass exposure is nearly universal in congenital cardiac surgery and introduces additional complexity. Hemodilution from circuit priming [15], hypothermia [16], inflammatory activation [17], and platelet dysfunction [18] all interact to shape postoperative bleeding risk. In neonates, bypass circuit prime may exceed native circulating volume, amplifying dilutional effects and transfusion exposure even before surgical bleeding occurs [15].

## 6. Challenges of Chest Tube Output as a Bleeding Measure

Chest tube output remains the most commonly used metric for defining postoperative bleeding in pediatric cardiac surgery [6]. It is readily available, continuously monitored, and easily expressed in mL/kg/hour, making it attractive for both clinical decision-making and research. However, reliance on chest tube drainage as a surrogate for true blood loss introduces substantial limitations.

The hemoglobin concentration of chest tube effluent is unknown and highly variable [19]. Drainage fluid represents a mixture of whole blood, serous fluid, residual cardiopulmonary bypass prime, irrigation solution, and mediastinal transudate. The relative contribution of each component changes over time, particularly in the early postoperative period when dilutional effects are greatest. The study titled “50 Shades of Red” found that surgeons performed poorly at matching hemoglobin concentrations to pre-filled drain samples, with only 30.4% (17/56) of surgeons correctly matching all six eligible hemoglobin concentrations [20]. As a result, a given volume of output does not reliably correspond to a defined quantity of red cell loss [21].

Furthermore, fixed numeric thresholds fail to account for patient size, surgical complexity, and hemodynamic tolerance.

Therefore, as a single-dimensional metric, chest tube output captures volume loss but not etiology or physiologic consequence. While chest tube output will likely remain a practical component of bleeding assessment, its limitations underscore the need for more nuanced, multidimensional definitions in pediatric cardiac surgery.

## 7. Inconsistency in Transfusion-Based Definitions

Transfusion exposure is frequently used as a surrogate marker of bleeding severity in pediatric cardiac surgery. Many studies define major bleeding by the volume of packed red blood cells, plasma, platelets, or cryoprecipitate administered within a specified postoperative time window [3,9,22]. At first glance, transfusion volume appears to offer a practical and quantifiable endpoint. However, transfusion-based definitions introduce substantial variability and conceptual limitations.

Transfusion decisions are influenced not only by blood loss but also by institutional protocols, clinician preference, laboratory interpretation, and patient-specific factors such as ventricular function, oxygenation strategy, and anticipated reintervention [23]. As a result, transfusion volume reflects both bleeding and management philosophy. Two patients with identical blood loss may receive markedly different transfusion volumes depending on local practice patterns.

## 8. Interventions vs. Outcomes in Bleeding Definitions

Similarly, a central issue in bleeding definitions is whether severity should be defined by the bleeding event itself or by the interventions that follow. Many definitions classify bleeding as “major” if it results in transfusion, escalation of vasoactive support, or return to the operating room for surgical hemostasis [6,22]. While these criteria are intuitively appealing, they risk conflating the underlying pathophysiology with clinician response.

Return to the operating room is frequently used as a marker of severe postoperative bleeding. Yet the threshold to re-explore varies substantially between cardiac surgeons and intensivists, and between institutions. Some surgical teams favor early re-exploration in the setting of persistent drainage, prioritizing rapid mechanical control of bleeding. Others may tolerate higher chest tube output if hemodynamics remain stable and laboratory parameters are reassuring, opting instead for continued observation or medical management. In certain contexts, clinicians may escalate pharmacologic therapy, including administration of coagulation factor concentrates or activated factor VII, rather than proceeding directly to reoperation. These divergent strategies reflect differences in training, experience, and institutional culture rather than intrinsic differences in bleeding severity.

Defining bleeding by the need for intervention is conceptually equivalent to defining appendicitis by whether an appendectomy was performed. The intervention reflects clinical judgment and contextual factors, rather than solely the biological severity of the disease.

## 9. VAD and ECMO Definitions

In contrast to the variability seen in postoperative cardiac surgery, mechanical circulatory support communities have made deliberate efforts to standardize bleeding definitions. In ventricular assist device (VAD) and extracorporeal membrane oxygenation (ECMO) populations, consensus definitions were developed to improve consistency in registry reporting and clinical research.

Within the VAD literature, the PediMACS registry, the pediatric arm of the Interagency Registry for Mechanically Assisted Circulatory Support (INTERMACS), represents a systematic attempt to standardize adverse event reporting in children on mechanical circulatory support [8]. Adverse events in PediMACS are categorized using a pre-specified dictionary of definitions derived by expert consensus, working in large part from the INTERMACS definitions and modified where necessary to be appropriate for pediatric patients. Major bleeding episodes are defined as bleeding episodes requiring (1) transfusion, (2) hospitalization, (3) surgery, or (4) resulting in death. However, the PediMACS bleeding definition, like those used in postoperative cardiac surgery, remains primarily intervention-anchored and does not incorporate physiologic or hemodynamic thresholds in a graded severity definition.

Within ECMO, the Extracorporeal Life Support Organization (ELSO) has established structured definitions to standardize complication reporting across its international registry [9]. ELSO definitions categorize bleeding events by site, clinical consequence, and associated interventions, allowing more consistent multicenter data aggregation and benchmarking.

More recently, the ECMO-CENTRAL consensus initiative has proposed refined, harmonized bleeding definitions to align research endpoints across trials and registries [10]. Within the ECMO-CENTRAL consensus definition, severe bleeding (Type 3) is defined as overt bleeding accompanied by at least two markers of physiologic or clinical compromise. Quantitative thresholds include sustained blood loss exceeding 5 mL/kg/hour for two or more hours, or more than 40 mL/kg within 24 h; in patients weighing more than 50 kg, equivalent criteria are greater than 250 mL/hour for two hours or more, or more than 2000 mL within 24 h. However, volume criteria alone are insufficient. Severe bleeding must also be associated with evidence of instability or organ compromise, such as hemodynamic deterioration not explained by other causes, escalation of vasoactive support, or the need for volume administration to maintain ECMO pump preload. Laboratory evidence of impaired oxygen delivery may also qualify, including an absolute hemoglobin decrease greater than 3 g/dL within 12 h accompanied by a lactate level above 3 mmol/L that is rising on serial measurements. Finally, bleeding that results in cardiac tamponade or compartment syndrome is classified as severe regardless of volume. This multidimensional approach integrates quantitative blood loss with physiologic impact, offering a more behavior-independent and clinically anchored definition of bleeding severity.

These definitions emphasize objective physiologic impact, attempt to separate bleeding severity from transfusion practice, and provide clearer operational criteria for multicenter studies.

The evolution of ELSO and ECMO-CENTRAL definitions demonstrates that harmonization is achievable even in highly complex, anticoagulated populations supported by extracorporeal circuits. Their experience offers a practical model for postoperative congenital cardiac surgery. Aligning surgical bleeding definitions with established ECMO definitions could promote continuity across the perioperative period, enhance comparability between supported and non-supported patients, and strengthen both quality improvement and clinical research efforts.

## 10. EMR Limitations and Data Capture Problems

Efforts to standardize bleeding definitions are further constrained by structural limitations in electronic medical record systems. Major U.S. platforms, such as Epic and Cerner, do not natively incorporate structured postoperative bleeding definitions into routinely captured data fields [24]. While institutions may develop local flowsheets to document bleeding severity, these tools are often not mapped in a standardized manner to institutional data warehouses. Consequently, they cannot be reliably extracted for quality improvement benchmarking, registry reporting, or multicenter research.

In practice, the only consistently structured variables available across systems are vital signs, hourly chest tube output, and routine laboratory values such as hemoglobin and lactate. Chest tube output reflects volume but does not account for the unknown proportion of true blood within the effluent [20]. Other clinically relevant manifestations of bleeding, such as oozing around central or arterial line sites, persistent soaking of surgical dressings, sanguineous endotracheal or nasogastric secretions, subcutaneous hematomas, or hemodynamic instability attributable to bleeding, are typically documented in narrative notes rather than discrete fields. Organ dysfunction scores such as PELOD-2, which anchor the BASIC definition, are similarly unavailable as discrete fields in most systems, in part because they require daily worst-value adjudication while excluding erroneous measurements. Because these elements are unstructured, they cannot be systematically queried or compared across centers.

Making these elements available would require several additions to the electronic medical record: automated calculations of baseline hemodynamics and proportional changes during suspected bleeding episodes, structured prompts quantifying the amount of blood in nasogastric and endotracheal tube secretions, and structured fields capturing the frequency and degree of dressing saturation. Even with this infrastructure, structured capture alone cannot resolve the question of causation. A patient who becomes hypotensive after a propofol bolus and who happens to have a single drop of blood in the nasogastric tube should not be classified as severe bleeding, because the hemodynamic change was not caused by the bleed. Adjudication of attribution will therefore remain a necessary step, performed by the bedside clinician in real time. Nonetheless, structured capture of the underlying variables and their mapping to institutional data warehouses would substantially reduce adjudication burden and enable automated extraction into multicenter registries. Piloting a harmonized definition within a single pediatric quality collaborative could allow iterative refinement before wider deployment.

Some investigators have attempted to identify bleeding events using administrative diagnostic codes. However, reliance on coding alone is inadequate. In one study, the sensitivity of diagnostic codes in identifying massive bleeding events was only 38%, demonstrating substantial under-detection of clinically significant bleeding [25]. This low sensitivity underscores the limitations of using billing or discharge codes as proxies for bleeding severity. Without structured integration of standardized bleeding definitions into EMR architecture, accurate surveillance and multicenter benchmarking will remain fundamentally constrained.

## 11. Why Standardization Matters

Standardization of postoperative bleeding definitions is essential for both quality improvement and scientific advancement. National quality collaboratives increasingly rely on benchmarked metrics to compare institutional performance, identify outliers, and drive targeted improvement initiatives [26]. Without a shared definition of bleeding, benchmarking becomes unreliable. Apparent differences in bleeding rates may reflect definitional variation rather than true differences in surgical technique, hemostatic management, or patient risk. This undermines the credibility of quality improvement efforts and limits the ability to identify best practices.

The implications extend to multicenter research. A recent publication examining bleeding during ECMO identified 69 distinct bleeding definitions across studies, resulting in wide heterogeneity in reported bleeding prevalence [27]. Such variability makes it difficult to determine the true burden of bleeding, compare outcomes across cohorts, or synthesize evidence through meta-analysis. When definitions vary, measured incidence becomes a function of criteria rather than biology.

The same risk exists in postoperative pediatric cardiac surgery. Without universally agreed-upon definitions, the field cannot accurately quantify the magnitude of bleeding, identify modifiable risk factors, or rigorously evaluate interventions designed to reduce bleeding. Standardization is therefore not a semantic exercise but a prerequisite for meaningful benchmarking, reproducible research, and the development of evidence-based strategies to minimize bleeding and improve outcomes.

## 12. Conclusions

Postoperative bleeding in pediatric cardiac surgery remains a clinically consequential yet incompletely defined entity. As outlined throughout this review, existing definitions vary widely across studies and institutions, often relying on chest tube output, transfusion exposure, or procedural intervention. Each of these approaches captures part of the bleeding spectrum but fails to provide a fully physiologic, developmentally calibrated, and behavior-independent definition. The resulting heterogeneity limits benchmarking, obscures true bleeding prevalence, complicates multicenter research, and impairs the interpretation of interventional trials.

**Figure 1:**
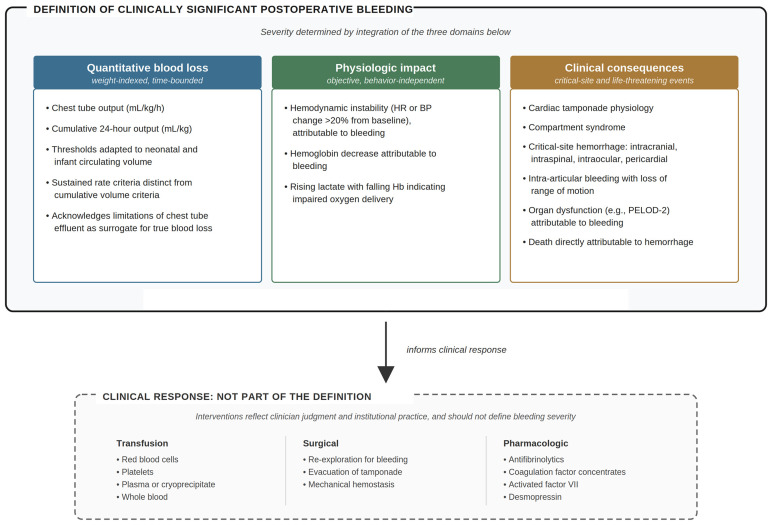
Proposed multidimensional framework for defining clinically significant postoperative bleeding in pediatric cardiac surgery. Bleeding severity is based on three domains: quantitative blood loss, physiologic impact, and clinical consequences. In contrast, transfusion, surgical, and pharmacologic responses are shown separately because they reflect management rather than the definition itself. HR, heart rate; BP, blood pressure; Hb, hemoglobin; PELOD-2, Pediatric Logistic Organ Dysfunction-2.

Experiences from harmonization efforts in ECMO and VAD populations demonstrate that consensus definitions, even if imperfect, can meaningfully strengthen comparability and research rigor. Pediatric cardiac surgery now faces a similar opportunity. A multidimensional definition anchored in objective blood loss, physiologic impact, and clinical consequence, while clearly distinguishing bleeding from the interventions used to treat it, would provide a more stable foundation for perioperative hemostasis management (Figure 1).

Standardization is not merely an academic exercise. It is essential to quantify the true burden of bleeding, evaluate strategies to minimize transfusion exposure, and ultimately improve outcomes for infants and children undergoing complex cardiac surgery.

## Figures and Tables

**Table 1:** Comparison of bleeding definitions across adult and pediatric definitions, including mechanical circulatory support.

Definition (Reference)/Target Population	Quantitative Blood Loss Criteria	Physiologic/Clinical Impact Criteria	Intervention-Based Criteria	Behavior-Independent?
Adult consensus definitions				
ISTH Major Bleeding, Schulman and Kearon, 2005 [3] Adults on anticoagulation (non-surgical)	Not quantified	Fatal bleeding OR Bleeding in a critical site: Intracranial Intraspinal Intraocular Pericardial Retroperitoneal Intramuscular with compartment syndrome OR Hemoglobin decrease ≥2 g/dL	Transfusion of 2 units of whole blood or packed red blood cells	Partial Critical-site and Hb criteria are behavior-independent; the transfusion criterion is clinician-driven
BARC Type 4, Mehran et al., 2011 [4] Adults undergoing coronary artery bypass grafting	Chest tube drainage ≥2 L within 24 h (not weight-indexed)	Perioperative intracranial hemorrhage within 48 h	Any of the following: Reoperation after sternal closure to control bleeding Transfusion ≥5 units of whole blood or packed red blood cells within 48 h	Partial Intracranial hemorrhage and chest tube volume are behavior-independent; reoperation and transfusion criteria are clinician-driven
TIMI Major Bleeding, Sabatine and Braunwald, 2021 [5] Adults receiving thrombolytic or antithrombotic therapy	Not quantified	Intracranial hemorrhage OR Hemoglobin decrease ≥5 g/dL OR Hematocrit decrease ≥15% (with or without an identified bleeding site)	Not included	Yes Anchored in intracranial hemorrhage and laboratory findings; no intervention-based criteria
Pediatric and mechanical circulatory support definitions				
Bercovitz et al., 2018 [6] Neonates and infants undergoing cardiac surgery with cardiopulmonary bypass	Chest tube output ≥7 mL/kg/h for ≥2 consecutive hours within the first 12 postoperative hours OR Chest tube output ≥84 mL/kg total within the first 24 postoperative hours	Not included	Surgical re-exploration for bleeding or cardiac tamponade physiology within the first 24 postoperative hours	Partial Volume criteria are behavior-independent; the re-exploration criterion is clinician-driven
BASIC, Nellis et al., 2019 [7] All critically ill children (applicable to the post-cardiac-surgery population)	Severe: quantifiable bleeding ≥5 mL/kg/h for ≥1 h Moderate: ≥1 but <5 mL/kg/h Minimal: <1 mL/kg/h	Severe bleeding requires any of the following: Organ dysfunction by PELOD-2 criteria within 24 h, attributable to bleeding Hemodynamic instability: HR increase >20% or BP decrease >20% from baseline, attributable to bleeding Hemoglobin drop >20% within 24 h attributable to bleeding Intraspinal bleeding with neurologic loss, intra-articular bleeding with decreased range of movement, or intraocular bleeding with impaired vision	Explicitly excluded; red cell transfusion and surgical interventions are not part of the definition	Yes
PediMACS/INTERMACS, Rosenthal et al., 2016 [8] Children supported by durable ventricular assist devices	Not quantified	Not quantified; death as a qualifying criterion	Major bleeding episode requiring any of: Transfusion Hospitalization Surgery Resulting in death	No (besides death) Primarily intervention-anchored
ELSO Registry, 2026 [9] Children supported by extracorporeal membrane oxygenation	Not expressed as continuous blood loss thresholds. The primary quantitative anchor is transfusion volume (see intervention column)	Events categorized by anatomic site: Intracranial hemorrhage Pulmonary bleeding Gastrointestinal bleeding Surgical site bleeding Cannulation site bleeding Tamponade due to bleeding Site diagnoses typically require confirmatory imaging (CT, US, MRI) or endoscopy	Packed red blood cell transfusion >20 mL/kg/24 h, or >3 units/24 h Endoscopic, radiologic, or surgical interventions required for confirmation of most site-specific events	No Primarily transfusion-anchored, with interventions embedded in site-specific event confirmation
ECMO-CENTRAL, Severe (Type 3), Alexander et al., 2024 [10] Children supported by extracorporeal membrane oxygenation	Sustained blood loss >5 mL/kg/h for ≥2 h, OR >40 mL/kg within 24 h For patients >50 kg: >250 mL/h for ≥2 h, OR >2000 mL within 24 h	Volume criteria alone are insufficient. Severe bleeding requires overt bleeding AND ≥2 markers of physiologic or clinical compromise: Hemodynamic deterioration not otherwise explained Escalation of vasoactive support Volume administration to maintain ECMO pump preload Hb decrease >3 g/dL within 12 h with lactate >3 mmol/L that is rising on serial measurements Cardiac tamponade or compartment syndrome classified as severe regardless of volume	Not required to meet definition	Partial Quantitative blood loss, Hb/lactate criteria, and critical consequences (tamponade, compartment syndrome) are behavior-independent. Hemodynamic deterioration is not quantitatively defined. Escalation of vasoactive support and volume administration to maintain pump preload are clinician-driven

## Data Availability

No new data were created or analyzed in this study.
